# Molecular epidemiology and multilocus genotyping of *Giardia duodenalis* in individuals attending major public hospitals in Shiraz, southwestern Iran: A public health concern

**DOI:** 10.1016/j.parepi.2024.e00354

**Published:** 2024-04-29

**Authors:** Ali Asghari, Farzad Mahdavi, Kambiz Karimi, Mohammad Reza Mohammadi, Laya Shamsi, Qasem Asgari, Mohammad Hossein Motazedian, Saeed Shahabi, Alireza Sadrebazzaz

**Affiliations:** a Children Growth Research Center, Research Institute for Prevention of Non-Communicable Diseases, Qazvin University of Medical Sciences, Qazvin, Iran; b Department of Medical Parasitology and Mycology, School of Medicine, Zanjan University of Medical Sciences, Zanjan, Iran; c Department of Medical Parasitology and Mycology, School of Medicine, Shiraz University of Medical Sciences, Shiraz, Iran; d Department of Bacteriology, Faculty of Medical Sciences, Tarbiat Modares University, Tehran, Iran; e Department of Pathobiology, Faculty of Veterinary Medicine, Urmia University, Urmia, Iran; f Department of Parasitology and Mycology, School of Medicine, Shiraz University of Medical Sciences, Shiraz, Iran; g Nanomedicine and Nanobiology Research Center, Shiraz University of Medical Sciences, Shiraz, Iran; h Department of Biology and Control of Disease Vectors, School of Health, Shiraz University of Medical Sciences, Shiraz, Iran; i Razi Vaccine & Serum Research Institute, Agricultural Research, Education and Extension Organization, Mashhad, Iran

**Keywords:** *Giardia duodenalis*, Prevalence, Assemblage, MLG, Shiraz, Iran

## Abstract

*Giardia duodenalis* is one of the most common causes of waterborne disease worldwide, and is often associated with outbreaks of diarrhea in areas with poor sanitation and hygiene. This study aimed to assess the prevalence and genetic diversity of *G. duodenalis* assemblages in individuals attending major public hospitals in Shiraz, southwestern Iran. From August 2022 to May 2023, a total of 614 stool samples from individuals were collected and initially examined for *G. duodenalis* cysts using parasitological techniques, sucrose flotation, and microscopy. Microscopy-positive samples were validated by SSU-PCR amplification of the parasite DNA. A multilocus genotyping (MLG) scheme, which focused on the triose phosphate isomerase (*tpi*) and the glutamate dehydrogenase (*gdh*) genes, was employed for genotyping purposes. *G. duodenalis* cysts were found in 7.5% (46/614) and 8.5% (52/614) of samples through microscopy and SSU-PCR, respectively. Successful amplification and sequencing results were obtained for 77.3% (17/22) and 45.5% (10/22) of the infected samples at the *tpi* and *gdh* loci, respectively. MLG data for the two loci were available for only five samples. Out of the 22 samples genotyped at any loci, 54.5% (12/22) were identified as assemblage A, while 45.5% (10/22) were identified as assemblage B. AII was the most predominant sub-assemblage identified [54.5% (12/22)], followed by BIII [27% (6/22)], discordant BIII/BIV [13.6% (3/22)], and BIV [4.5% (1/22)]. In the present study, no assemblages suited for non-human animal hosts (e.g., C—F) were detected. This suggests that the transmission of human giardiasis in Shiraz is primarily anthroponotic. Further molecular-based analyses are necessary to confirm and expand upon these findings. These analyses will also help determine the presence and public health importance of the parasite in environmental samples, such as drinking water.

## 1 Introduction

*Giardia duodenalis* (syn. *Giardia lamblia*, *Giardia intestinalis*) is a parasite that infects the small intestine of mammals, including humans. *G. duodenalis* is a globally distributed parasitic protozoan with a prevalence of 0.4–7.5% in developed countries and 8–30% in developing countries (Hashemi-Hafshejani et al., 2022; Heyworth, 2016). *G. duodenalis* infections are caused by ingesting cysts in contaminated food or water. Asymptomatic *G. duodenalis* infections are common in humans and usually clear within weeks without treatment (Rafiei et al., 2020). Asymptomatic infections can lead to malabsorption syndrome, characterized by poor growth in children in developing countries (Mahdavi et al., 2022; Pouryousef et al., 2023). Symptomatic disease causes gastrointestinal symptoms such as diarrhea, discomfort, flatulence, nausea, and bloating (Mahdavi et al., 2021). The *G. duodenalis* complex is composed of eight genotypes, referred to as assemblages A-H, that are morphologically identical but have diverse molecular lineages. *G. duodenalis* assemblages A and B infect humans and various mammals (Asghari et al., 2022b; Asghari et al., 2022a). While, assemblages C—H are typically found in dogs and other canids (C, D), hoofed livestock (E), cats (F), rodents (G), and marine mammals (H) (Asghari et al., 2023; Zajaczkowski et al., 2021). Nevertheless, recent evidence suggests that livestock-only circulating assemblages (i.e., E) may also infect humans, suggesting some assemblages have less strict host-specificity, potentially enabling transmission from non-human mammals to humans (Dixon, 2021; Fu et al., 2022). An allozyme examination specified four sub-assemblages within assemblages A and B (AI–AIV and BI–BIV), of which AI, AII, BIII, and BIV have been particularly recognized in humans. Following nucleotide sequence and phylogenetic analysis have confirmed sub-assemblages AI–AIII within assemblages A, with AI being isolated mostly from animals, whereas AII is primarily determined in humans. Additionally, AIII has mainly been reported in wild mammals (e.g., deer), with only two human cases reported recently (Hashemi-Hafshejani et al., 2022; Seabolt et al., 2022). Moreover, multilocus sequence typing (MLST) has distinguished 9–12 subtypes/genotypes at each of the individual loci within the three main sub-assemblages A. However, the analysis of genetic loci sequences has not found distinct sub-assemblages within assemblage B, likely due to high sequence diversity unsupported by bootstrap analysis. *G. duodenalis* assemblages A and B infect various mammalian hosts, including humans. Hence, these infections are of zoonotic significance (Costache et al., 2020; Klotz et al., 2022; Song et al., 2021).

Genetic markers such as small subunit ribosomal RNA (SSU-rRNA), glutamate dehydrogenase (*gdh*), triosephosphate isomerase (*tpi*), and b-giardin (*bg*) genes are frequently utilized to identify *G. duodenalis* in various hosts (Asghari et al., 2024), enabling the identification of genetic diversity and population dynamics among *G. duodenalis* assemblages (Köster et al., 2021). The SSU-rRNA gene is a highly conserved and multicopied locus, which makes it a potential substitute for identifying and distinguishing *G. duodenalis* assemblages. However, it is less useful in determining genetic diversity within assemblages due to its conserved nature and short amplified fragments in most PCR assays. On the other hand, the single-copy *tpi*, *bg*, and *gdh* loci are more sensitive in detecting genetic variation and categorizing *G. duodenalis* populations at the sub-assemblage and genotype levels. However, these loci are not considered potential candidates for identifying *G. duodenalis* in clinical settings. Although there is agreement on the ability of these loci to genetically classify different *G. duodenalis* assemblages, conflicting results have been reported regarding the efficacy of a single locus in distinguishing *G. duodenalis* populations into assemblages and sub-assemblages. Thus, in order to enhance accuracy, a numeric multilocus genotyping (MLG) procedure was introduced, which involves the simultaneous analysis of at least two genes (*tpi*, *bg*, and *gdh*) (Dan et al., 2019; Hashemi-Hafshejani et al., 2022; Heng et al., 2022; Köster et al., 2021; Oh et al., 2021; Rafiei et al., 2020).

Hence, due to the importance of giardiasis in humans, especially in immunocompromised patients and children under 5 years of age, and the lack of extensive multilocus genotyping study of *Giardia* among the people of Shiraz city, the present study aims to determine the prevalence, distribution of assemblages, and investigate the genetic diversity between human isolates of *G. duodenalis* among people referring to public hospitals in Shiraz city.

## 2 Materials and methods

### 2.1 Ethics approval

The study protocol was reviewed and approved by the Ethics Committee of Shiraz University of Medical Sciences (Approval No. IR.SUMS.REC.1400.460), Shiraz, Fars Province, Iran.

### 2.2 Study area

**Fig. 1:**
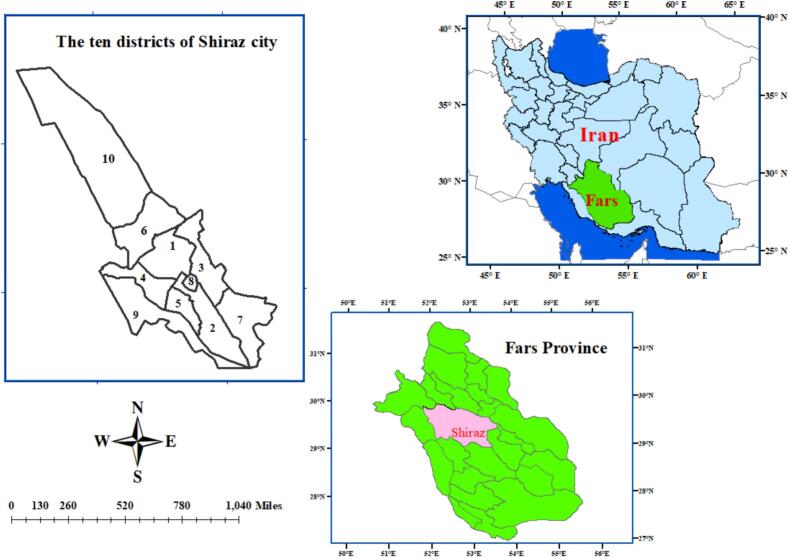
Map of Iran, Fars Province and Shiraz city showing sampling locality of the humans in the present study.

This is a descriptive cross-sectional study that investigated multilocus genotyping, assemblage distribution, and phylogeny of *G. duodenalis* infection in individuals attending major public hospitals in Shiraz at 29.5926 N latitude and 52.5836 E longitude between August 2022 and May 2023 (Fig. 1). Shiraz is a metropolis in Iran and the capital of Fars Province in the southwest of the country. According to the census 2021, the population of Shiraz metropolis was 1,955,500 people. Shiraz is the fourth largest and most populous city in Iran and the most populous city in the south of the country. This city is located in the central part of Fars Province, at an altitude of 1486 m above sea level and in the mountainous region of Zagros with a mild climate.

### 2.3 Sample collection and *G. duodenalis* identification

According to the arrangements made with the officials and experts of the parasitology department of Shahid Beheshti, Saadi, Namazi, and Hafez hospitals in Shiraz, 614 stool samples were randomly collected from people who were referred to laboratories of these hospitals. Direct microscopy using formalin-ether concentration was used for screening all stool samples (Rafiei et al., 2020). After microscopy examination, the sucrose-flotation method was conducted to isolate the cysts of *G. duodenalis* from positive samples. In brief, almost 5 g of stool was blended with 50 ml of distilled water, sifted by means of a four-layer gauze, and centrifuged at 1000 ×*g* for 5 min. Following the supernatant deletion, the residue was then mixed with 30 ml of distilled water and added to a 15 ml cold sucrose solution (1 M). After centrifuging at 800 ×*g* for 5 min, the top two layers with *G. duodenalis* cysts were moved to a new falcon tube and the volume was carried to 50 ml with distilled water. After three centrifugations at 1000 ×*g* for five min, a portion of the pellet was examined under a light microscope at 400 × magnification. The remaining sediment was stored at −20 °C for future molecular analysis.

### 2.4 DNA extraction

To facilitate cyst rupture and parasite DNA extraction, fecal pellets were subjected to five freeze-thaw cycles comprising 5 min in liquid nitrogen and 5 min in a boiling water bath. Genomic DNA was straightforwardly extricated employing a QIAamp DNA Stool Mini Kit (QIAgen, Hilden, Germany), according to the manufacturer's instruction. DNA was eluted in 100 mL of distilled water and prepared DNA was located at −20 °C prior to utilize for polymerase chain reaction (PCR).

### 2.5 Molecular identification of *G. duodenalis*

All fecal samples were re-checked using molecular analyses after microscopic examination. The parasite's presence was initially confirmed using a nested PCR protocol to amplify a ∼ 130-bp fragment of the SSU-rRNA gene (Gillhuber et al., 2013). Thus, primary and secondary reactions were conducted utilizing the outer primer pair RH11 and RH4, and the inner primer set GiarF and GiarR, respectively. The reaction mixture contained 2 μl of template DNA for both the primary and secondary PCR reactions. Cycling conditions were the same for the first and second steps of PCR, with primary denaturation at 95 °C for 3 min, followed by 35 cycles of amplification (denaturation at 95 °C for 30 s, annealing at 55 °C for 30 s), with a final extension of 7 min at 72 °C.

Positive *G. duodenalis* specimens at SSU-PCR were afterward analyzed utilizing a multilocus genotyping (MLG) method according to the amplification of partial sequences of the *gdh* and the *tpi* genes. To amplify a ∼ 432-bp fragment of the *gdh* gene, a semi-nested PCR was accomplished (Read et al., 2004). The protocol contained the outer primer set GDHeF and GDHiR, and the inner primer set GDHiF and GDHiR. Both PCR reaction mixtures included 5 μl of template DNA. Both the primary and secondary PCRs were performed as follows: an initial denaturation step of 95 °C for 3 min, followed by 35 cycles of 95 °C for 30 s, 55 °C for 30 s, and 72 °C for 60 s, with a final extension of 72 °C for 7 min. Regarding the *tpi* gene (Sulaiman et al., 2003), a ∼ 530-bp fragment was amplified employing the outer primer pair AL3543 and AL3546, and the inner primer pair AL3544 and AL3545. The primary PCR conditions included an initial denaturation phase at 94 °C for 5 min followed by 35 cycles of 94 °C for 45 s, 50 °C for 45 s, and 72 °C for 60 s, with a final extension of 72 °C for 10 min. The conditions for the secondary PCR were identical to the primary PCR.

All aforementioned PCR reactions were performed in a FlexCycler2 PCR Thermal Cycler (Analytik Jena, Jena, Germany). PCR mixtures (25 μL) comprised 12.5 μL of 2× Master Mix RED already consisting Taq DNA polymerase (Ampliqon-Biomol, Hamburg, Germany), 200 nM of each forward and reverse primer, and nuclease-free water. Negative (no DNA template) and positive (known *G. duodenalis* PCR-positive sample) controls were routinely contained in all PCR steps. Secondary PCR products were investigated by electrophoresis on 1.5% agarose gels (Sinacolon, Tehran, Iran) and visualized after ethidium bromide staining.

### 2.6 Sequencing and phylogenetic analyses

Received *gdh* and *tpi* amplicons of the right size were instantly dispatched for bidirectional sequencing employing the related internal primer pairs at Microsynth AG (Balgach, Switzerland). Raw sequences were visually checked utilizing the free software Chromas version 2.1 as quality control and to identify the existence of single nucleotide polymorphisms (SNPs) including double peaks. Acquired consensus sequences were evaluated with those formerly deposited at the National Centre for Biotechnology Information (NCBI) using the BLAST tool (http://www.ncbi.nlm.nih.gov/blast). Designation of assemblages and sub-assemblages was conducted by sequence alignment using ClustalW in MEGA X (www.megasoftware.net). A phylogenetic investigation was conducted on the sequences acquired in the present study at the *gdh* and *tpi* loci and earlier published sequences of human origin recovered from GenBank utilizing the Neighbor-Joining (NJ) procedure. Evolutionary connections were computed by the Kimura-2-parameter model in MEGA X. The trustworthiness of these trees was estimated by using the bootstrap method with 1000 pseudoreplicates. Regarding haplotype analyses, genetic diversity was measured based on haplotype diversity (Hd) and nucleotide diversity (π). Values for the numbers of polymorphic sites, parsimony informative sites, haplotype frequencies, and the average number of nucleotide differences among sequences were estimated. These genetic diversity values were computed using the software DnaSP v5.0 (Xu et al., 2015). The Median-joining haplotype network of *G. duodenalis* sequences was built by PopART v1.7 software (Leigh and Bryant, 2015). Representative sequences at the *gdh* and *tpi* loci generated in the present survey were deposited in GenBank under accession numbers OR488681-OR488690 and OR488693-OR488709.

## 3 Results

### 3.1 Microscopy and PCR-based prevalence of *G. duodenalis*

Based on formalin-ether concentration and saline/iodine wet mount examination of 614 fecal samples, 46 specimens (7.5%) were found to be infected with *G. duodenalis* by microscopy. In return, molecular analysis with SSU-rRNA gene revealed more sensitivity and detected 52 cases (8.5%).

### 3.2 Multilocus genotyping, sequencing, and assemblage distribution of *G. duodenalis*

**Fig. 2:**
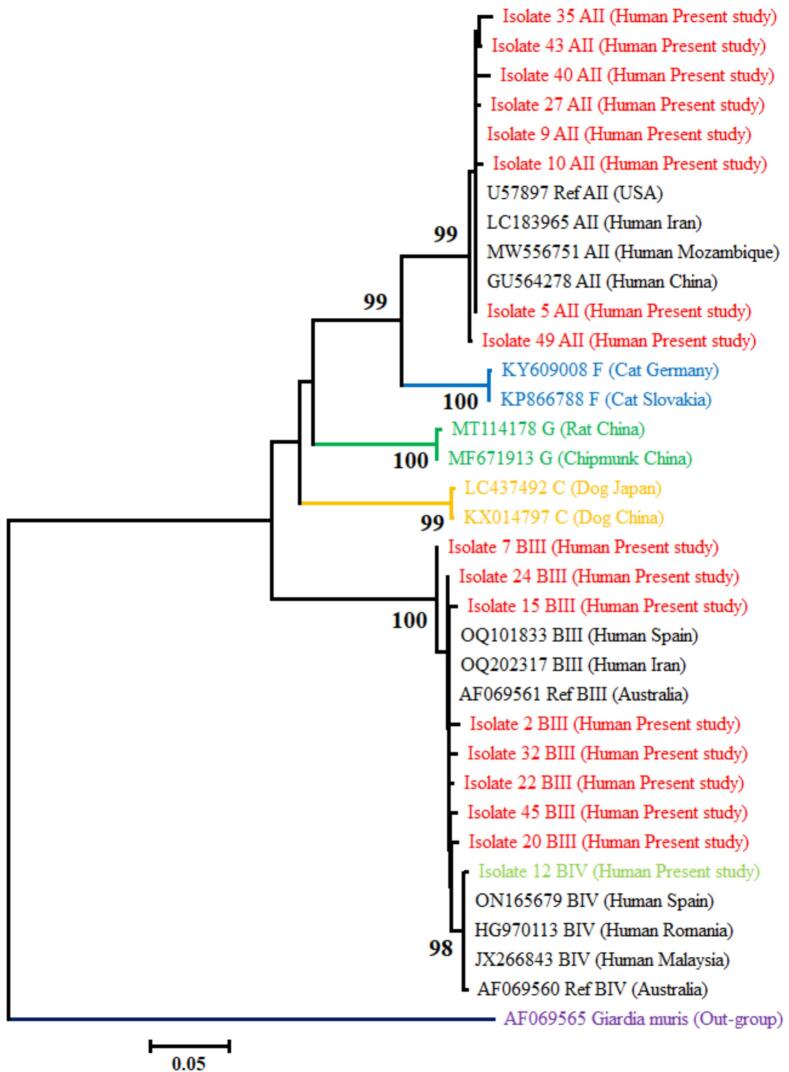
The phylogenetic tree was constructed from the sequence of positive cases of human G*. duodenalis* in the current study and sequences in GenBank based on the *tpi* gene using the Neighbor-Joining model.

**Fig. 3:**
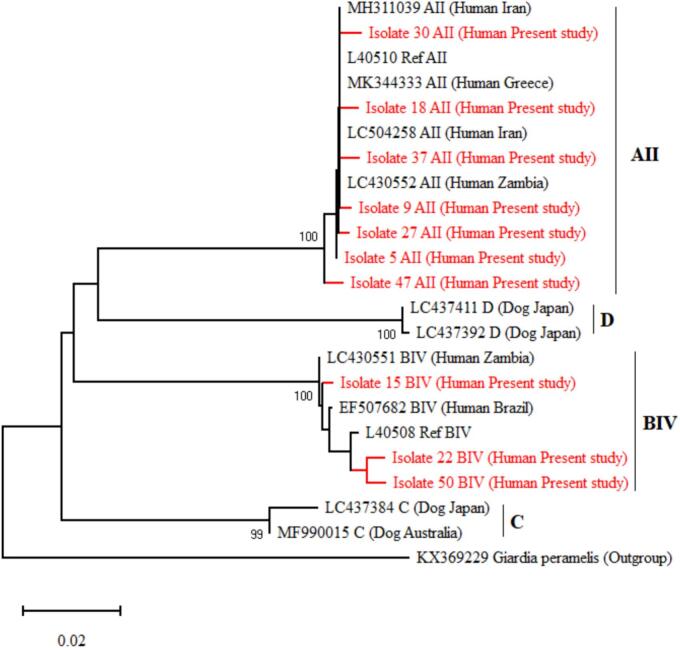
The phylogenetic tree derived from the positive cases of human *G. duodenalis* in this study and sequences from GenBank based on the *gdh* gene using the Neighbor-Joining model.

Out of 52 molecular positive samples, 22 positive samples were randomly selected and evaluated for the amplification of ∼530 and ∼ 432 bp fragments of *tpi* and *gdh* genes by Nested-PCR. Among the 22 examined samples, PCR amplification and successful sequencing by *tpi* and *gdh* genes were 77.3% and 45.5%, respectively. The phylogenetic analysis using Neighbor-joining method and Kimura 2-parameter model demonstrated two assemblages (A and B) and three sub-assemblages (AII, BIII, and BIV) among infected people (Fig. 2, Fig. 3). The assemblage/sub-assemblages of all 22 examined samples was characterized by at least one gene locus. Accordingly, 54.5% (12/22 samples) of the positive cases belonged to sub-assemblage AII, 27% (6/22 samples) to sub-assemblage BIII, 13.6% (3/22 samples) were identified as sub-assemblage BIII/BIV and 4.5% (1/22 samples) were detected as sub-assemblage BIV (Table 1). No A + B mixed infections, nor host-specific assemblages of canine, feline, or livestock (C—F) origin were detected.

**Table 1:** Distribution of *G. duodenalis* assemblages/sub-assemblages among 22 positive human samples based on *tpi* and *gdh* genes.

Isolate ID	Gene loci		Assigned assemblage
	*gdh*	*tpi*	
2	–	BIII	BIII
5	AII	AII	AII
7	–	BIII	BIII
9	AII	AII	AII
10	–	AII	AII
12	–	BIV	BIV
15	BIII/BIV	BIII	BIII
18	AII	–	AII
20	–	BIII/BIV	BIII/BIV
22	BIII/BIV	BIII	BIII
24	–	BIII	BIII
27	AII	AII	AII
30	AII	–	AII
32	–	BIII	BIII
35	–	AII	AII
37	AII	–	AII
40	–	AII	AII
43	–	AII	AII
45	–	BIII/BIV	BIII/BIV
47	AII	–	AII
49	–	AII	AII
50	BIII/BIV	–	BIII/BIV

Out of the 10 *gdh* sequences identified, seven were assigned to the sub-assemblage AII and three were discordant BIII/BIV results. Of the five AII sequences, one (OR488681) showed 100% identity with a previously described reference sequence (GenBank accession number L40510), and the remaining four varied from 1 to 4 SNPs with L40510. The three BIII/BIV sequences had 3 and 4 SNPs (Table 2). Out of the 17 *tpi* sequences identified, eight were assigned to the sub-assemblage AII, six to the BIII, one to the BIV, and three were discordant BIII/BIV results. Of the eight AII sequences, one (OR488698) showed 100% identity with a previously described reference sequence (GenBank accession number U57897), and the remaining sixteen varied from 2 to 5 SNPs with U57897. The six BIII, three BIII/BIV, and one BIV sequences had 1 and 6 SNPs (Table 2).

**` 2:** Genetic diversity, nucleotide variations, and accession numbers of *G. duodenalis* sequences isolated from the present study and reference samples registered in the GenBank based on *tpi* and *gdh* genes.

Locus	Assemblage	Sub-assemblage	Isolate ID	Ref. sequence	SNVs	Access no.
*gdh*	A	AII	5	L40510	None	OR488681
			9	L40510	C20Y, A426T	OR488682
			18	L40510	C19A, C222G, A342C, A411G	OR488683
			27	L40510	C10T	OR488684
			30	L40510	T98G, C183G, A370C, G375C	OR488685
			37	L40510	A104T, A305T, T323G, T367A	OR488686
			47	L40510	A7T, C156G, T384A	OR488687
	B	BIII/BIV	15	L40508	T118C, C208T, T322C, A373G	OR488690
			22	L40508	C191T, C209T, A395G	OR488688
			50	L40508	T72C, C165G, C192T, C210T,	OR488689
*tpi*	A	AII	5	U57897	None	OR488698
			9	U57897	C71M, G489C	OR488694
			10	U57897	C23A, G155C, G297T, T267Y	OR488700
			27	U57897	A24C, G431A	OR488697
			35	U57897	A28G, T84C, A368C, G447A	OR488693
			40	U57897	G97C, T225A, C298G, G399C, C503G	OR488695
			43	U57897	G15C, T302Y, G446A, G479T	OR488696
			49	U57897	G84Y, A299Y, T355C, T493G	OR488699
	B	BIII	2	AF069561	C33T, C99A, G112C	OR488701
			7	AF069561	T10G, G36A, T252C, T282Y	OR488702
			15	AF069561	T294C, A364G,	OR488704
			22	AF069561	G313C	OR488705
			24	AF069561	C3G, A135R, G305R, C336Y	OR488708
			32	AF069561	A105G, C121T	OR488709
		BIV	12	AF069560	A178T, A392G	OR488703
		BIII/BIV	20	AF069561	A38C, G46T, C111Y, A139R, C141Y, C275Y	OR488707
			45	AF069561	C71G, C92T, C108Y, C171Y, G177R, G399R	OR488706

### 3.3 Haplotype analyses

**Fig. 4:**
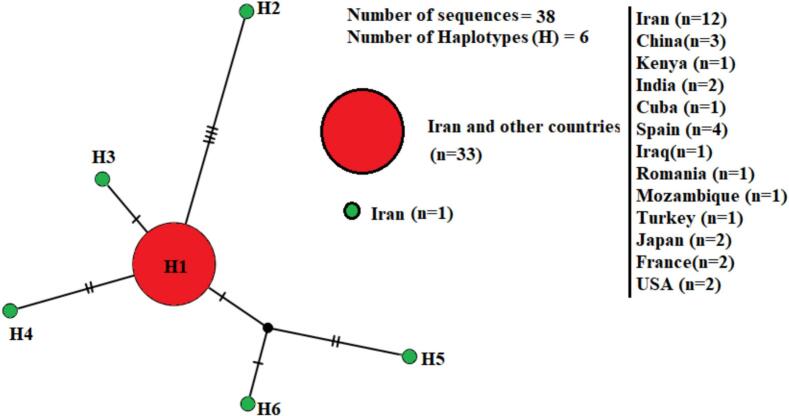
Median-joining haplotype network of *G. duodenalis* assemblage A sequences based on triose-phosphate isomerase (*tpi*) gene. Circle size is relative to haplotype frequency (n); the black circle represents extinct or unsampled haplotypes. Hatch marks on the line represent mutational steps between haplotypes. Haplotype colors represent the geographic locations of haplotypes as indicated in the right corner of the figure (green=Iran, red=Iran, and other countries).

Regarding *tpi*-based sequences of assemblage A, 11 sites were variable (polymorphic) and one site was parsimony informative, resulting in the identification of six haplotypes from 38 *G. duodenalis* sequences isolated from Iran and various countries. Accordingly, average number of the nucleotide differences (k), haplotype diversity (Hd), and nucleotide diversity (π) were 0.629, 0.249, and 0.00136 respectively. Of the six obtained haplotypes, five were from Iran which were not shared with any of the investigated regions. Whereas, only one haplotype was shared among Iran and other countries. Of note, in the present study, haplotype 1 (H1) occurred in the most sequences of *G. duodenalis* with the most prevalence from Iran (Fig. 4).

**Fig. 5:**
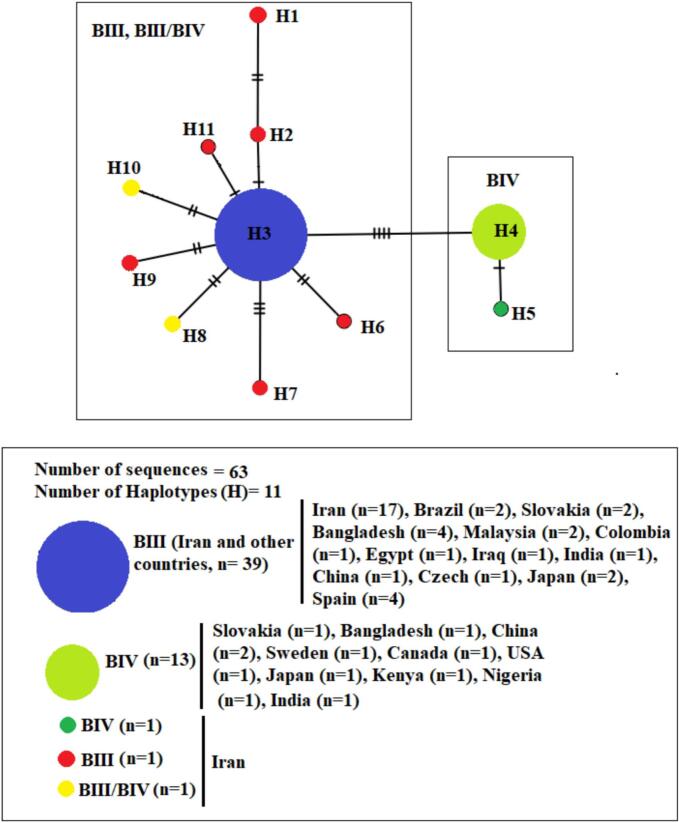
Median-joining haplotype network of *G. duodenalis* assemblage A sequences based on triose-phosphate isomerase (tpi) gene**.** Circle size is relative to haplotype frequency (n). Hatch marks on the line represent mutational steps between haplotypes. Haplotype colors represent geographic locations of haplotypes as indicated in the figure.

Concerning *tpi*-based sequences of assemblage B, 20 sites were variable (polymorphic) and five site was parsimony informative, leading to the detection of 11 haplotypes from 63 *G. duodenalis* sequences isolated from Iran and various countries. The average number of nucleotide differences (k), haplotype diversity (Hd), and nucleotide diversity (π) were 0.629, 0.552, and 0.0043 respectively. From 11 identified haplotypes, nine were not shared among Iran and other countries. In terms of sub-assemblage BIII, haplotype 3 (H3) occurred in the most sequences of *G. duodenalis* (*n* = 39) and shared among Iran and other countries. Moreover, from 10 haplotypes of Genotype B which found in Iran, nine haplotypes were special to Iran. From two haplotypes of BIV (*n* = 14) only one haplotype (H5, n = 1) found in Iran, while the haplotype H4 with the most frequently (*n* = 13) shared between other countries. No BIV haplotype was shared between Iran and other countries (Fig. 5).

## 4 Discussion

Giardiasis is a major cause of diarrhea, particularly among children under the age of five in developing nations (Kiani et al., 2020). In Iran, the prevalence of giardiasis in humans has been documented to range from 5% to 23% in different regions of the country (Abasian et al., 2013). Among domestic animals, goats have a prevalence rate of 5–16%, while sheep have rates of 6–20%, and cattle have a rate of 4% (Asghari et al., 2022a; Rafiei et al., 2020). Nevertheless, there is a dearth of molecular epidemiological studies utilizing the MLG system in Iran. The available molecular data on giardiasis in Iran are limited to a few studies that utilized PCR-RFLP and nested-PCR techniques to examine specific loci such as *gdh*, *tpi*, and/or *bg* (Pestehchian et al., 2012; Tappeh et al., 2014). Previous investigations of human giardiasis in Iran, specifically in Shiraz, primarily relied on microscopy, with molecular methods being used only for single-locus analyses (Bahrami et al., 2017; Kasaei et al., 2018; Mahmoudi et al., 2020; Rayani et al., 2014). Molecular findings in the country indicate that assemblage A and sub-assemblage AII are the primary genetic variants of *G. duodenalis* found in the Iranian population (Hashemi-Hafshejani et al., 2022; Mirrezaie et al., 2019). To our knowledge, this is the first investigation employing MLG schemes based on *tpi* and *gdh* loci in *G. duodenalis*-infected individuals in Shiraz, the capital of Fars Province, southwestern Iran.

In this study, *G. duodenalis* cysts were detected in 7.5% (46/614) and 8.5% (52/614) of samples using microscopy and SSU-PCR, respectively. This prevalence rate is considered moderate when compared to global reports on human giardiasis (Dixon, 2021). To further investigate, MLG and sequencing were conducted on 22 out of the 52 positive *G. duodenalis* isolates obtained from individuals referred to Saadi, Shahid Beheshti, Hafez, and Motahari hospitals in Shiraz. This analysis aimed to identify the infective assemblages/sub-assemblages and assess the genetic diversity among the relevant sequences. Our findings revealed that individuals infected with *G. duodenalis* in Shiraz were infected with assemblages A and B, consistent with global reports of human giardiasis (Feng and Xiao, 2011). While a previous study (Ryan and Cacciò, 2013) indicated that assemblage B is more common than A worldwide, in line with our results, several studies in Iran (Babaei et al., 2008; Hooshyar et al., 2017; Rayani et al., 2014), Turkey (Tamer et al., 2015), Iraq (Turki et al., 2015), Syria (Skhal et al., 2017), Saudi Arabia (Al-Mohammed, 2011), Egypt (Helmy et al., 2009), Thailand (Traub et al., 2009), Italy (Cacciò et al., 2002), Czech Republic (Lecová et al., 2018), and Ethiopia (Gelanew et al., 2007) have reported a predominance of assemblage A. The discrepancies may result from the geographical distribution, the populations studied as well as the different genetic and molecular tools employed (Hashemi-Hafshejani et al., 2022; Rafiei et al., 2020), as the impact of loci is evident in the assemblage B results in *tpi* [52.9% (9/17)] compared to *gdh* [30% (3/10)] genes in the present study. Moreover, the amplification rates of the genetic loci mentioned were not uniform. Most primers could detect about 60% of the *tpi* genes and 40–60% of the *gdh* genes (Feng and Xiao, 2011). This could explain the differing amplification rates of *tpi* (77.3%) and *gdh* (45.5%) reported in the present study, which are consistent with previous findings (Feng and Xiao, 2011; Huey et al., 2013). In this study, three sub-assemblages, namely AII, BIII, and BIV, were identified among the individuals investigated in Shiraz. These findings shed light on the distribution and prevalence of different sub-assemblages of *G. duodenalis* in the human population. Notably, the AII sub-assemblage is closely associated with humans, while various sub-assemblages of B are primarily linked to zoonotic infections (Rafiei et al., 2020; Sprong et al., 2009).

In the current study, out of 22 positive samples tested with MLG schemes, 17 samples (77.5%) identified *G. duodenalis* by the *tpi* gene, 10 samples (45.5%) by the *gdh* gene, and 5 samples (22.7%) by both *tpi* and *gdh* genes. Of note, on a per-gene basis, some samples were not amplified, did not have optimal sequencing, or were not assigned to a specific assemblage/sub-assemblage; as mentioned in a previous study, this phenomenon may be due to the various factors including insufficient amount or suboptimal quality of parasite DNA, or inefficient removal of PCR inhibitors during the DNA extraction, purification process, and/or mixed infections (Cacciò and Ryan, 2008; Feng and Xiao, 2011; Huey et al., 2013). Based on the haplotyping findings, Iran exhibited high haplotype diversity for assemblage A, with six haplotypes identified among 17 examined sequences. Conversely, only one common haplotype was found among 21 sequences from various countries, highlighting the distinctiveness of Iran in terms of haplotype distribution for this gene. Furthermore, a shared haplotype was observed between Iran and other countries, indicating potential genetic connections. Additionally, Iran showed a significant presence of assemblage B haplotypes, highlighting the importance of Giardia parasites in the area and suggesting Iran as a potential origin for these assemblages, particularly BIII. Of note, the haplotyping aspect of the present study might not align entirely with statistical approaches, given the noticeable disparity in the number of Iranian sequences in comparison to other nations. Augmenting the sequences from other countries may lead to a potentially increased count of haplotypes.

Overall, our findings indicate that humans are likely a possible source of infection and person-to-person transmission probably takes place in Shiraz. However, the main limitation of this hypothesis is the limited data on non-human giardiasis in Iran. To address this issue, it is necessary to conduct extensive molecular analyses to identify the specific type of *Giardia* infection in humans, companion animals, and livestock that live together or exist in the same area. Additionally, the analysis should include environmental *G. duodenalis* isolates.

## 5 Conclusion

This is the first MLG study to genetically identify human isolates of *G. duodenalis* in Shiraz, the capital of Fars Province, southwestern Iran. Our MLG analysis findings showed a higher occurrence of assemblage A compared to assemblage B in the human community residing in Shiraz. The absence of sub-assemblage AI in the studied individuals supports the hypothesis that most human giardiasis in Shiraz is acquired through anthroponotic sources. Overall, further advanced molecular-based epidemiological investigations are necessary to validate the extent of the aforementioned findings.

## Acknowledgements and funding

The current study was a part of the PhD thesis of Ali Asghari, financed by the Vice-Chancellor for Research of Shiraz University of Medical Sciences (Grant No: 21882). Hereby, we would like to express our gratitude and appreciation for the comprehensive support of this center.

## CRediT authorship contribution statement

**Ali Asghari:** Data curation, Investigation, Methodology, Writing – original draft, Writing – review & editing. **Farzad Mahdavi:** Investigation, Writing – original draft. **Kambiz Karimi:** Investigation, Methodology. **Laya Shamsi:** Investigation. **Qasem Asgari:** Investigation, Methodology. **Mohammad Hossein Motazedian:** Conceptualization, Project administration, Supervision. **Saeed Shahabi:** Investigation, Methodology. **Alireza Sadrebazzaz:** Investigation.

## Declaration of competing interest

The authors declare no potential conflicts of interest with respect to the research, authorship, and/or publication of this article.

## Data Availability

The datasets used and/or analyzed during the current study are available in the online version.
